# From companion animals to patients: interspecies lessons in neuroendocrine oncology

**DOI:** 10.3389/fvets.2026.1830861

**Published:** 2026-06-19

**Authors:** Fernanda Cristina Poscai Ribeiro, Rodrigo A. Maioral, Victoria Cristina Mendonça Fujimoto, Gabriele Drigo Galan, Jaqueline Bastos-Lebold, Gary Chris Fillmore, Heloisa P. Soraes, Erika Said Abu Egal

**Affiliations:** 1 Department of Internal Medicine, University of Western São Paulo (UNOESTE), São Paulo, Brazil; 2 Biorepository and Molecular Pathology, Huntsman Cancer Institute, University of Utah (UU), Salt Lake City, UT, United States; 3 Department of Oncology, Huntsman Cancer Institute, University of Utah (UU), Salt Lake City, UT, United States; 4 Graduate Program in Veterinary Medicine in the Coastal Environment, Metropolitan University of Santos (UNIMES), São Paulo, Brazil

**Keywords:** animal therapies, comparison, gastroenteropancreatic neoplasms, human therapies, neuroendocrine tumors

## Abstract

Neuroendocrine tumors (NETs) are rare heterogeneous neoplasms that arise in multiple organs of humans and companion animals and can produce diverse hormonal syndromes or remain clinically silent, leading to delayed diagnosis. Given shared environments and conserved neuroendocrine morphology across species, companion animals represent an attractive yet underexplored model for comparative oncology. This literature review searched PubMed through December 2025 and qualitatively synthesized evidence on the epidemiology, macroscopic and histologic features, biomarkers, clinical presentation, imaging modalities, treatment, and prognosis of neuroendocrine tumors in humans, dogs, and cats. Collectively, this review indicates that neuroendocrine tumors in dogs and cats recapitulate many key biological and clinical features of human NETs and therefore hold meaningful translational potential, particularly for functional pancreatic, hepatic, and intestinal tumors. However, substantial gaps in veterinary epidemiologic surveillance, biomarker validation, imaging standardization, and prospective therapeutic evaluation currently limit full comparative integration. Addressing these gaps through coordinated, multicenter comparative studies and harmonized reporting frameworks could enhance diagnosis and management in veterinary patients while advancing spontaneous animal models for human NET research.

## 1 Introduction

Neuroendocrine tumors (NETs) encompass a heterogeneous group of neoplasms arising from neuroendocrine cells, specialized cells that combine properties of nerve and endocrine cells, capable of producing biogenic amines and peptide hormones. Because neuroendocrine cells are distributed throughout many organs, NETs may originate virtually anywhere in the body, although the most common primary sites in humans are the gastrointestinal tract, pancreas, and lungs (1, 2).

Despite their relative rarity compared to more common epithelial malignancies, NETs hold substantial clinical significance. Their biological behavior is extremely variable, ranging from indolent, slow-growing tumors to aggressive high-grade neoplasms, and they may remain asymptomatic for prolonged periods, leading to delayed diagnosis. Furthermore, many NETs are functional, secreting hormones or amines that can result in distinct clinical syndromes (e.g., hypoglycemia, Zollinger-Ellison syndrome, carcinoid syndrome), whereas non-functionals may manifest only when organ mass effect or metastasis produces clinical signs (3, 4).

Companion animals such as dogs and cats frequently share the same household environment as humans, breathing the same air, drinking the same water, using the same spaces for rest and activity, and thus are exposed to the same environmental agents (5).

Also, given the morphological and histogenetic similarity of NETs across mammalian species, there is a compelling rationale for comparative oncology approaches. However, the interspecies translational potential remains poorly explored: the paucity of systematic comparative studies limits understanding of how differences in physiology, tumor biology, and clinical presentation may influence disease progression and therapeutic response across species (6, 7).

Therefore, a comparative analysis of NETs in humans and domestic animals may provide valuable insights into tumor biology, identify shared or divergent mechanisms, and potentially inform both veterinary and human medicine.

## 2 Methodology

This literature review aimed to compare the presentation of neuroendocrine tumors (NETs) in domestic animals and in humans. We performed a literature search and qualitative synthesis of data on epidemiology, histopathological characteristics, clinical signs, imaging findings, diagnostic methods, treatment, and prognosis.

The electronic database PubMed was searched from database inception through December 2025 using a predefined search strategy. No language or study-design restrictions were applied. The primary search string for animal studies was:

(“Insulinoma” OR “islet cell tumor” OR “islet cell neoplasm” OR “beta-cell tumor” OR “neuroendocrine tumor” OR “neuroendocrine tumor” OR “neuroendocrine carcinoma” OR “neuroendocrine neoplasm” OR “neuroendocrine carcinoid” OR “carcinoid tumor” OR “gastrinoma” OR “Zollinger-Ellison” OR “glucagonoma” OR “somatostatinoma” OR “VIPoma” OR “vasoactive intestinal peptide-secreting tumor” OR “gastroenteropancreatic neuroendocrine neoplasm” OR “GEP-NEN” OR “pancreatic endocrine tumor” OR “pancreatic neuroendocrine tumor”) AND (“Dog” OR “canine” OR “Cats” OR “feline” OR “Animals, Domestic”)

For human studies, we used the same disease-related terms (“Insulinoma” OR “islet cell tumor” OR “islet cell neoplasm” OR “beta-cell tumor” OR “neuroendocrine tumor” OR “neuroendocrine tumor” OR “neuroendocrine carcinoma” OR “neuroendocrine neoplasm” OR “neuroendocrine carcinoid” OR “carcinoid tumor” OR “gastrinoma” OR “Zollinger-Ellison” OR “glucagonoma” OR “somatostatinoma” OR “VIPoma” OR “vasoactive intestinal peptide-secreting tumor” OR “gastroenteropancreatic neuroendocrine neoplasm” OR “GEP-NEN” OR), but omitted the animal terms and, where appropriate, supplemented the query with additional keywords specific to the target subsection (“treatment,” “biomarkers,” “histology,” “macroscopy,”“epidemiology,” “imaging,” “prognosis,” or “clinical findings”).

Titles and abstracts from the search results were screened to remove clearly irrelevant records. Full texts of potentially eligible articles were retrieved and assessed against the inclusion criteria, defined as original research articles, case series, and review papers reporting data on at least one of the predefined domains addressed in the subsections of this review. No restrictions were applied regarding language or year of publication in the search strategy.

Studies were excluded if they were not focused on neuroendocrine tumors, did not involve humans or companion animals, were editorials and conference abstracts. Two independent reviewers (FCPR and RAM) initially screened titles and abstracts, followed by full-text assessments. In case of disagreements, a third reviewer (ESAE) was designated, though no conflicts occurred. It was previously established that, in cases of missing information or unavailability of the full text, the corresponding authors would be contacted by email for clarification. However, this approach was not necessary.

In total, 146 studies met the inclusion criteria and were incorporated into the qualitative synthesis.

Data relevant to the predefined domains were synthesized narratively, with emphasis on similarities and differences between species in a final table.

## 3 Epidemiology

### 3.1 Epidemiology of NETs in humans

Neuroendocrine neoplasms (NENs) comprise a heterogeneous and relatively uncommon group of malignancies (Table 1), accounting for roughly 2% of cancers and affecting fewer than 200,000 individuals in the United States, a prevalence that qualifies them as an orphan disease (8). Incidence has risen over recent decades, currently estimated at approximately 5.86 per 100,000 per year, a trend largely attributed to heightened clinical awareness and improved diagnostic capabilities. The gastrointestinal tract (62–67%) and the lung (22–27%) are the predominant primary sites, and between 12 and 22% of patients are present with metastatic disease. Most NENs arise sporadically, although associations with Multiple Endocrine Neoplasia type 1 (MEN1) and familial aggregation have been documented; smoking and alcohol do not appear to increase NEN risk (9).

**Table 1:** Epidemiology of NETs (humans vs. animals).

Parameter	Humans	Animals (Dogs/Cats)
Disease Burden	~2% of cancers <200,000 in the US Qualified as an orphan disease (8)	Rare No population-level registries Data from case series and pathology reviews (15, 16)
Incidence	5.86/100,000 per year 6.4-fold increase from 1973 (1.09/100,000) to 2012 (6.98/100,000) (9, 10)	Formal incidence not established in most regions (17)
Primary Sites	GI tract (62–67%) Lung (22–27%) Unknown primary (0.84/100,000) (9–11)	PanNENs are the most documented pNETs are considered the commonest in dogs (15–17)
Insulinoma Incidence	1–4 cases per million people per year Most common pancreatic NET (12, 13)	Studies suggest a higher frequency in dogs compared to humans Formal population-level data not established (17)
Metastatic Presentation at Diagnosis	12–22% of patients (9)	frequently evident at diagnosis (15, 17, 18)
Gender Distribution	Female: 52.7% Male: 47.3% (10)	No definitive sex predisposition confirmed (17, 18)
Age at Presentation	Mean age: 58 ± 15 years (GEP-NETs) (11)	Limited feline data (15, 17, 18)
Breed/Population Predisposition	Not applicable	Medium to large dog breeds, Boxers, Terriers, and Retrievers are overrepresented No definitive breed predisposition confirmed (17, 18)
Hereditary Syndromes	MEN1 association (5–10% of insulinomas) (12, 13)	Not clearly established hereditary syndromes (17, 18)
Risk Factors	Familial aggregations for MEN1 No established risk factors (12, 13)	Risk determinants remain lacking (17, 18)
Survival Trends	Improved over the past 3 decades Poorly differentiated tumors still have poor outcomes (14)	Veterinary evidence is fragmentary (15, 17, 18)

A large retrospective series of 64,971 NET cases reported a female proportion of 52.7% (34,233 cases) and an age-adjusted incidence that rose 6.4-fold from 1973 (1.09/100,000) to 2012 (6.98/100,000), with increases observed across sites, stages and grades; site-specific incidence was highest for gastroenteropancreatic locations (3.56/100,000), followed by lung (1.49/100,000) and unknown primary (0.84/100,000) (10). In another study, 43,751 patients were diagnosed with GEP-NETs between 1975 and 2015 (mean age 58 ± 15 years), illustrating the substantial case volumes captured by population registries (11).

Specific subtypes remain rare: insulinomas in humans occur at an estimated 1–4 cases per million persons per year and are the most common pancreatic NET; 5–10% of human insulinomas are associated with MEN1 (12, 13). Overall survival for NETs has improved over the past three decades, although outcomes for poorly differentiated tumors remain poor (14).

### 3.2 Epidemiology of NETs in animals

By contrast, NET epidemiology in domestic animals (principally dogs and, less frequently, cats) is sparsely characterized (Table 1). Available data derive largely from retrospective case series and pathology reviews rather than from population-level registries, limiting precise estimates of incidence and prevalence (15). Certain presentations, such as gallbladder neuroendocrine neoplasms in dogs, are described as among the rarest hepatobiliary neoplasms in that species (16).

Among veterinary reports, pancreatic neuroendocrine neoplasms (PanNENs), and notably functional tumors such as insulinomas, are the most frequently documented entities (15). Insulinoma is considered the commonest pancreatic NET in dogs, although a formal population-level incidence has not been established, some evidence suggests a higher relative frequency in dogs than in humans (17). The literature is dominated by case reports and small series that emphasize clinical features and outcomes, with few large cohorts addressing breed predisposition or other risk factors. Most published canine cases involve medium to large breeds, and Boxers, terriers, and retrievers are often overrepresented; nevertheless, no definitive breed or sex predisposition has been confirmed, and data on other risk determinants remain lacking (17, 18).

### 3.3 Comparison

Epidemiologically, NETs in humans are increasingly well-defined owing to comprehensive cancer registries and improved diagnostic ascertainment, with a documented rise in incidence across sites and grades and demonstrable improvements in survival for many patients. In contrast, NET occurrence in animals remains poorly quantified: veterinary evidence is fragmentary. Both species most commonly manifest pancreatic NETs in the form of insulinoma. Also, hereditary syndromes analogous to human MEN1 are not clearly established in veterinary cohorts. Overall, while human NET epidemiology benefits from systematic surveillance and robust incidence data, animal NETs require focused, population-level investigation to define their true burden and risk factors.

## 4 Macroscopic, histological, and biomarker characteristics of neuroendocrine tumors

### 4.1 Canine neuroendocrine gallbladder carcinoma

**Figure 1:**
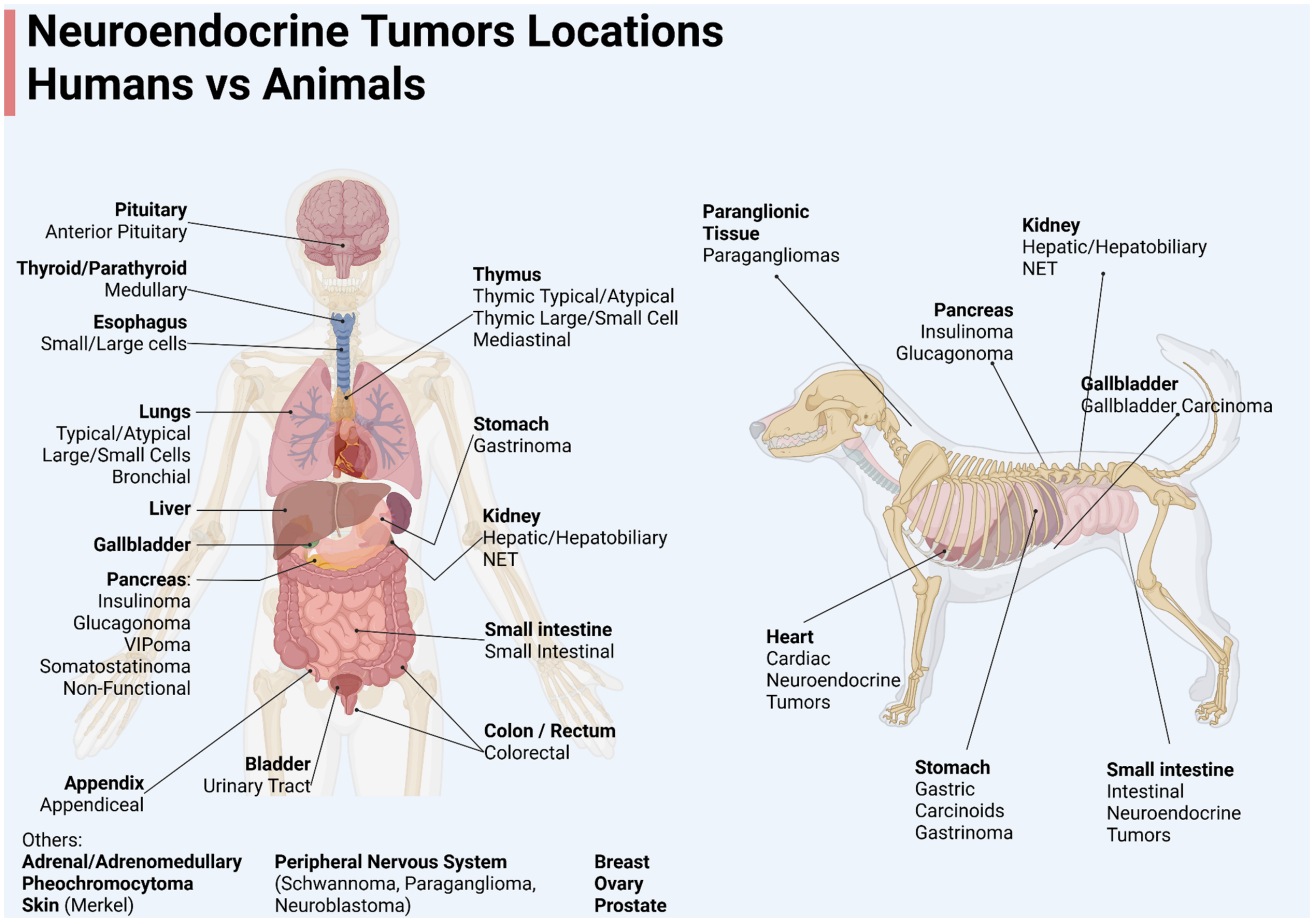
Neuroendocrine tumors locations compared between humans and animals. Created by: BioRender.com.

From a morphological standpoint, macroscopic alterations observed in the gallbladder include a solid, firm intravesicular mass of variable size, frequently associated with gallbladder wall thickening, inflammatory changes, and thickened bile content. Microscopically, the neoplasm exhibits a solid growth pattern supported by a delicate fibrovascular stroma and is composed of round to polygonal cells arranged in differentiated architectural patterns, such as nests, rosettes, or solid cords. The neoplastic cells show granular cytoplasm and enlarged nuclei, marked nuclear pleomorphism, high mitotic activity, and, in several cases, vascular invasion (16, 19) (Table 2). Also, Figure 1 provides an effective visual representation of anatomical tumor distribution.

**Table 2:** Macroscopic, histological, and biomarker characteristics of NETs (animals only).

Characteristic	Insulinoma	Glucagonoma	Gastrinoma	Gallbladder	Pancreatic NET
Macroscopic Features	Nodular, solitary or multiple; spherical, small Yellow to dark red color Non-encapsulated Firmer than the surrounding parenchyma (21–23)	Small nodules (<2 cm) Well-circumscribed and firm Whitish color (23–25)	Solitary or multiple Variable dimensions Firm Frequently metastatic at diagnosis (23, 26, 27)	Solid, firm intravesicular mass of variable size Gallbladder wall thickening Inflammatory changes Thickened bile content (16, 19)	Solid neoplasm Wide size variation Softer than the surrounding pancreatic tissue (20)
Location in the Pancreas	Right or left lobes Less frequent in the body (21–23)	Parenchyma; no specific site predilection (23–25)	Throughout the pancreas; often metastatic to the liver and lymph nodes at diagnosis (23, 26, 27)	N/A (hepatobiliary origin)	Mainly in the body and tail of the pancreas, but may involve the entire organ (20)
Histological Pattern	Polygonal and well-defined border cells, pale eosinophilic granular cytoplasm Thin fibrous stroma (21–23)	Aggregates and sheets of small cells, poorly defined cytoplasmic borders, and trabecular arrangements Delicate collagenous stroma (23–25)	Rounded cells arranged in nests and cords Rosette or pseudorosette formation Fibrous stroma Clear, finely granular cytoplasm, oval nuclei, low mitotic activity (23, 26, 27)	Solid growth pattern; nests, rosettes, or solid cords Delicate fibrovascular stroma Vascular invasion in several cases Round to polygonal cells, granular cytoplasm, enlarged pleomorphic nuclei, high mitotic activity (16, 19)	Small round neoplastic cells; occasional rosette formation, nests of variable size Thin fibrous stroma Minimal vascularity Scant cytoplasm, argyrophilic granules, small, round, hyperchromatic nuclei (20)
Functional Secretory Products	Insulin Proinsulin (primary) (47)	Glucagon (primary) May co-secrete gastrin (24, 50–52)	Gastrin may co-secrete other hormones (27, 54–56)	Variable May be non-functional or produce ectopic hormones (16, 19)	IGF-II in some cases (paraneoplastic hypoglycemia) (40, 41)

### 4.2 Pancreatic neuroendocrine tumors

Pancreatic neuroendocrine tumors in animals are classified as adenomas or carcinomas, whereas in humans, they are classified as pancreatic neuroendocrine tumors when well differentiated, or pancreatic neuroendocrine carcinomas when poorly differentiated (15).

In general, pancreatic neuroendocrine tumors are solid neoplasms located mainly in the body and tail of the pancreas, although they may involve the entire organ. They exhibit wide size variation, ranging from very small lesions to large masses, and are usually softer than the surrounding pancreatic tissue. Histologically, the pancreatic mass is composed predominantly of small round neoplastic cells, with minimal participation of epithelial cells. These cells are arranged in nests of variable size separated by a thin fibrous stroma and display small, round, hyperchromatic nuclei, scant cytoplasm with argyrophilic granules positive on Grimelius staining, and occasional rosette formation (20).

### 4.3 Insulinomas

In general, canine insulinomas are present as nodular tumors, solitary or occasionally multiple, spherical, small, and ranging in color from yellow to dark red. These neoplasms are non-encapsulated and are usually firmer than the surrounding pancreatic parenchyma, being separated from adjacent tissue by a thin layer of fibrous tissue. The tumors are composed of polygonal cells with well-defined cell borders and pale eosinophilic granular cytoplasm. Most of these neoplasms are in the right or left pancreatic lobes and are less frequently found in the body of the pancreas (21–23).

### 4.4 Glucagonomas

These tumors are composed of aggregates and sheets of small neoplastic cells with poorly defined cytoplasmic borders, separated by a delicate collagenous stroma. In certain areas, the cells are arranged in trabecular patterns. Macroscopically, the lesions appear as small nodules, generally less than 2 cm in diameter, well-circumscribed, firm in consistency, and whitish in color within the pancreatic parenchyma (23–25).

### 4.5 Gastrinomas

These tumors may be present as solitary or multiple lesions with variable dimensions. They are generally firm on palpation, a feature attributed to increased fibrous connective tissue within the stroma. Frequently, metastatic disease is already evident at the time of diagnosis. Histologically, the tumors are composed of rounded cells arranged in nests and cords, with rosette or pseudorosette formation, clear and finely granular cytoplasm, oval nuclei, and low mitotic activity, surrounded by a fibrous stroma. This tumor type typically exhibits aggressive behavior, with lymphatic invasion and metastasis to the liver and lymph nodes, while maintaining similar cellular characteristics between the primary tumor and metastatic lesions (23, 26, 27).

### 4.6 Gastric carcinoids

Macroscopically, these neoplasms appear as well-demarcated nodules within the gastric wall. Microscopically, the tumor consists of rounded neoplastic cells arranged in nests supported by a delicate fibrovascular stroma. The nuclei range from round to oval and vary in size. Occasional mitotic figures are observed, along with cytoplasm exhibiting fine stippling or a pink vacuolated appearance (28, 29).

### 4.7 Hepatic and hepatobiliary neuroendocrine tumors

From a morphological perspective, hepatic and hepatobiliary neuroendocrine tumors typically present macroscopically as firm masses, dark red to brown in color, multilobulated and poorly demarcated, infiltrating the hepatic parenchyma. Histologically, they are composed of thick cellular cords with multiple cell layers, as well as acinar arrangements or packets of polygonal cells supported by a delicate fibrovascular stroma. The neoplastic cells display central or eccentric, round, finely stippled nuclei and abundant, finely granular eosinophilic cytoplasm (30, 31).

### 4.8 Intestinal neuroendocrine tumors

Microscopically, the resected tumor exhibits cells arranged in nests and cords separated by a delicate fibrovascular stroma. The neoplastic cells are round to polyhedral and display eosinophilic granular cytoplasm. The nuclei range from small to moderate in size, with coarsely aggregated chromatin. In addition, frequent mitotic activity is observed (32, 33).

### 4.9 Paragangliomas

Microscopic examination reveals neoplastic cells with granular cytoplasm arranged in nests or cellular groups, forming the characteristic Zellballen pattern, separated by a thin fibrovascular stroma. Macroscopically, tumor appearance varies according to location; however, in general, it is presented as a multilobulated mass (34).

### 4.10 Cardiac neuroendocrine tumors

These tumors may exhibit an irregular nodular configuration, white to grayish coloration, and solid consistency, with small hemorrhagic foci distributed throughout some areas of the tumor mass. Microscopically, a population of predominantly oval cells is observed, with focal areas of polygonal cells, showing oval nuclei with finely granular chromatin. Marked anisocytosis and anisokaryosis are evident, along with megalokaryosis, prominent nucleoli, and increased mitotic activity (35).

### 4.11 Biomarkers

Immunohistochemistry (IHC) in neuroendocrine tumors primarily aims to confirm the neuroendocrine differentiation of neoplastic cells, complementing macroscopic and microscopic findings. Most neuroendocrine tumors are characterized by the expression of classical immunohistochemical markers, particularly synaptophysin, chromogranin A, and neuron-specific enolase. However, due to variability in the expression of these markers in canine tumors, the use of a combined antibody panel is important (36–38).

In human oncology, NETs are routinely stratified using standardized grading systems based on proliferative activity, particularly the mitotic count and the Ki-67 labeling index, as classification for gastroenteropancreatic NETs. This framework categorizes tumors into G1, G2, and G3 (134). In contrast, veterinary pathology traditionally classifies NETs in dogs and cats into adenomas and carcinomas based primarily on morphological criteria and evidence of invasion or metastasis (15).

Incorporating proliferation markers such as Ki-67 into veterinary diagnostic workflows has been proposed. Preliminary veterinary studies have demonstrated the feasibility of Ki-67 assessment in neuroendocrine carcinomas of the canine mammary gland, for exemple, suggesting correlations with biological behavior similar to those observed in humans (37). Despite this potential, challenges currently limit the routine adoption of human-style grading systems in veterinary oncology. These include the cost and availability of immunohistochemical assays, variability in laboratory protocols, lack of standardized cut-off values for Ki-67 interpretation across species, and the absence of large multicenter validation studies.

## 5 Clinical findings

### 5.1 Animals

#### 5.1.1 Pancreatic neuroendocrine tumors

Pancreatic neuroendocrine tumors in dogs and cats are uncommon and frequently present with nonspecific, progressive clinical signs. Affected animals typically exhibit gastrointestinal and systemic manifestations, including vomiting, anorexia or hyporexia, abdominal distension, weight loss, lethargy, and ascites. Physical examination often reveals dehydration, cachexia, pale mucous membranes, and hypothermia in advanced cases. Laboratory abnormalities may include azotemia, anemia, hypoalbuminemia, and altered liver enzyme activities. In cats, metastatic dissemination is commonly present at diagnosis, with clinical deterioration associated with hepatic and peritoneal involvement (20–21, 40).

A subset of functional pancreatic NETs is associated with paraneoplastic hypoglycemia due to ectopic secretion of insulin-like growth factor II (IGF-II). Clinically affected dogs present with episodic weakness, collapse, tremors, seizures, and altered mentation, often triggered by fasting, exercise, stress, or excitement, and characterized by recurrent neuroglycopenic events with inappropriately low or normal insulin concentrations (40, 41).

#### 5.1.2 Insulinomas

Insulinomas are characterized clinically by recurrent hypoglycemia and predominant neurological manifestations. Typical clinical signs in dogs include intermittent seizures, collapse, generalized weakness, posterior paresis, ataxia, tremors, and behavioral changes. These episodes are commonly precipitated by fasting, physical exertion, excitement, or stress, while interictal periods may be clinically normal, particularly in early-stage disease (42–44).

Prolonged or severe hypoglycemia may cause irreversible cerebral damage, progressing to coma and death. In large case series, the most frequent presenting signs include weakness, epileptic seizures, and altered consciousness or behavior, with many dogs showing neurological deficits such as obtundation, reduced withdrawal reflexes, and absent menace responses (45).

Rare ectopic insulin-secreting tumors have been reported and should be suspected in cases of persistent hypoglycemia without detectable pancreatic masses (21). Paraneoplastic syndromes may occur, including superficial necrolytic dermatitis, characterized by hyperkeratotic, crusted lesions involving the muzzle, eyelids, ventral abdomen, pinnae, and paw pads (46).

Feline insulinomas, although rarely reported, show clinical manifestations similar to those of dogs, including weakness, collapse, ataxia, and seizure activity, with temporary clinical improvement following glucose administration (47).

Canine insulinomas exhibit a particularly aggressive clinical course, with a high frequency of metastatic disease compared with human cases, contributing to systemic clinical deterioration (48).

Additional reports describe severe hypoglycemic crises accompanied by excessive salivation, vomiting, recumbency, and histologic evidence of cortical neuronal injury (49).

#### 5.1.3 Glucagonomas

Glucagon-secreting neuroendocrine tumors are predominantly associated with distinctive dermatologic and metabolic clinical syndromes. The most characteristic manifestation is superficial necrolytic dermatitis (also referred to as necrolytic migratory erythema or hepatocutaneous syndrome), which presents painful, erythematous, crusted, and ulcerative skin lesions affecting the abdomen, inguinal region, distal limbs, paw pads, and mucocutaneous junctions. Systemic signs typically include lethargy, anorexia, vomiting, and progressive weight loss (24, 50–52).

Many animals develop diabetes mellitus and concurrent hepatopathy, with clinical evidence of chronic liver dysfunction. Clinical progression is often chronic and debilitating, and progressive deterioration occurs despite supportive management. Similar dermatologic syndromes may occur in the absence of identifiable glucagon-secreting tumors, particularly in association with primary hepatic failure (53).

#### 5.1.4 Gastrinomas

Gastrin-secreting tumors induce a hypersecretory acid state, resulting in severe gastrointestinal ulceration and erosive disease. Clinically, affected dogs and cats most commonly manifest persistent or intermittent vomiting, anorexia, weight loss, lethargy, diarrhea, and ptyalism. Regurgitation may be present in cases of severe esophagitis caused by chronic acid reflux (27, 54–56).

Gastrointestinal hemorrhage, manifested as melena or hematemesis, and laboratory findings indicating hypoalbuminemia are commonly observed (57).

Feline gastrinomas have been described in patients with chronic vomiting, anorexia, lethargy, and weight loss, often with imaging evidence of duodenal or pancreatic lesions (58–60).

Canine gastrinomas, including rare duodenal forms, are associated with persistent vomiting, anorexia, diarrhea, hepatobiliary abnormalities, and progressive clinical deterioration (61). Zollinger–Ellison syndrome has been reported in cats presenting with vomiting, weight loss, lethargy, diarrhea, and multiple duodenal ulcers (62).

#### 5.1.5 Gastric carcinoids

Gastric carcinoids are rare neoplasms that generally manifest with chronic, nonspecific gastrointestinal signs such as persistent vomiting, anorexia, weight loss, and lethargy. After complete surgical excision, some animals remain clinically stable, whereas others show progressive disease (28).

In dogs with advanced disease, additional clinical findings may include weakness, ataxia, cough, anemia, hypoalbuminemia, and ultrasonographic evidence of gastric and metastatic lesions (29).

#### 5.1.6 Hepatic and hepatobiliary neuroendocrine tumors

Primary hepatic and hepatobiliary neuroendocrine tumors typically present with nonspecific systemic signs, including anorexia, weight loss, vomiting, lethargy, and hepatomegaly. Extrahepatic forms are frequently accompanied by icterus and biochemical evidence of cholestasis or hepatocellular injury (30, 31).

Paraneoplastic endocrine syndromes may occur, including hypoglycemia with seizure activity and, more rarely, ectopic hormone secretion resulting in hypercortisolism and persistent hypokalemia, leading to episodic myopathy and muscle weakness (63).

Gallbladder neuroendocrine neoplasms in dogs are most frequently present with gastrointestinal bleeding and hepatobiliary signs. Typical clinical manifestations include vomiting, hematemesis, melena, progressive weight loss, abdominal pain, and lethargy. Laboratory abnormalities often consist of anemia and increased liver enzyme activities (19, 64–67).

#### 5.1.7 Intestinal neuroendocrine tumors

Intestinal neuroendocrine tumors are most commonly present with signs of gastrointestinal hemorrhage and chronic enteropathy. Affected animals frequently develop melena, hematemesis, chronic anemia, lethargy, anorexia, vomiting, intermittent diarrhea, and progressive emaciation. Advanced disease is often complicated by metastatic spread, contributing to systemic clinical decline (32, 33).

#### 5.1.8 Paragangliomas

Paragangliomas are rare neuroendocrine neoplasms arising from paraganglionic tissue and may be clinically silent or associated with signs of excess catecholamine. Reported clinical manifestations include systemic hypertension, weakness, and biochemical evidence of mild hepatobiliary dysfunction. Some cases present as incidental findings during imaging for unrelated conditions (68, 69).

#### 5.1.9 Cardiac neuroendocrine tumors

Cardiac neuroendocrine tumors typically produce clinical signs secondary to mass effect on cardiac chambers. Dogs commonly present with cough, tachypnea, exercise intolerance, and weakness. Progressive right-sided congestive heart failure may develop, with clinical evidence of ascites and respiratory compromise (70).

### 5.2 Clinical findings (humans)

Neuroendocrine tumors (NETs) in humans represent a heterogeneous group of neoplasms capable of producing hormones, biogenic amines, or being non-functioning; clinical presentation varies considerably depending on tumor type, functional status, and location (71, 72).

#### 5.2.1 Pancreatic neuroendocrine tumors

Pancreatic NETs account for a small fraction of pancreatic neoplasms, but their incidence appears to be rising. Many are nonfunctioning, thus remain clinically silent until they reach a size sufficient to produce mass effects or metastasize, often leading to late diagnosis (73). When functioning, pNETs may secrete a variety of hormones, giving rise to defined clinical syndromes (74).

Insulinoma: Typically presents with episodic fasting hypoglycemia (the classical Whipple’s triad), leading to neuroglycopenic symptoms (confusion, blurred vision, headache, weakness) and autonomic manifestations such as tremor, sweating, palpitations, anxiety, in severe cases, seizures or coma may occur (75).

Gastrinoma (Zollinger-Ellison syndrome): Characterized by excessive acid secretion due to hypergastrinemia, leading to severe or refractory peptic ulcers, gastroesophageal reflux, abdominal pain, diarrhea, and possible malabsorption (76).

Glucagonoma: Frequently presents with the hallmark skin lesion Necrolytic Migratory Erythema (NME) in 55–90% of patients, coupled with weight loss, hyperglycemia or overt diabetes, anemia, glossitis, stomatitis, cheilitis, diarrhea or altered bowel habits, and general cachexia. Venous thromboembolic events and hypoaminoacidemia are also common laboratory or clinical findings (77).

Other less common functional pNETs: Include tumors secreting vasoactive intestinal peptide (VIPomas), presenting with profuse watery diarrhea, dehydration, hypokalemia, achlorhydria (the WDHA syndrome), or somatostatinomas, associated with diabetes mellitus, steatorrhea, cholelithiasis, and weight loss (78, 79).

#### 5.2.2 Carcinoid syndrome

Another significant subgroup of NETs arises from the gastro-entero-pancreatic tract (GEP-NETs). Among these, some tumors secrete serotonin or other vasoactive amines, giving rise to the classic Carcinoid Syndrome. Typical clinical manifestations include episodic flushing (commonly of the head and neck, sometimes precipitated by stress, certain foods, alcohol, or hot beverages), diarrhea, abdominal cramping, and wheezing, often associated with malabsorption or bronchospasm (80, 81).

In advanced or metastatic disease (commonly involving the liver), fibrosis of right-sided heart valves may develop, leading to valvular heart disease (e.g., tricuspid regurgitation, pulmonic stenosis) (82).

#### 5.2.3 General findings

Because many NETs are indolent and slow-growing, nonspecific systemic symptoms are frequently reported, particularly in nonfunctioning or poorly differentiated neoplasms. These may include fatigue, weight loss, anorexia, abdominal discomfort or pain, and signs related to metastatic spread (e.g., hepatomegaly, ascites). Nonfunctioning gastrointestinal NETs may also remain asymptomatic for long periods, with clinical detection often resulting from complications related to mass effect or incidental imaging (83).

### 5.3 Comparative analysis

Several human NET syndromes closely mirror those described in veterinary cases (Table 3). For example, Functional pNETs in humans (insulinoma, gastrinoma, glucagonoma) correspond to similar entities in dogs and cats, with overlapping clinical presentations (hypoglycemia/neuroglycopenic signs, ulcer disease, diarrhea, dermatitis, weight loss, etc.). Also, nonfunctioning NETs in both humans and animals share the challenge of late diagnosis due to silent growth and nonspecific symptoms.

**Table 3:** Clinical findings comparison (humans vs. animals).

Syndrome/tumor type	Humans	Animals (Dogs/Cats)
Insulinoma	Whipple’s Triad: Neuroglycopenic symptoms: confusion, blurred vision, headache, weakness Autonomic symptoms: tremor, sweating, palpitations, anxiety Severe cases: seizures, coma (75)	Predominant neurological manifestations Intermittent seizures, collapse, generalized weakness, posterior paresis, ataxia, tremors, behavioral changes Precipitated by fasting, exercise, excitement, stress Interictal periods may be clinically normal in early stages Severe/prolonged hypoglycemia: epileptic seizures, altered consciousness/behavior, neurological deficits Risk of irreversible cerebral damage, coma, death Hypoglycemic crises with excessive salivation, vomiting, recumbency, histologic cortical neuronal injury (21, 42–49)
Gastrinoma (Zollinger-Ellison)	Excessive acid secretion, severe/refractory peptic ulcers Gastroesophageal reflux Abdominal pain Diarrhea, possible malabsorption (76)	Persistent or intermittent vomiting Anorexia, weight loss, lethargy Diarrhea, ptyalism Regurgitation (severe esophagitis from acid reflux) GI hemorrhage: melena or hematemesis Hypoalbuminemia (27, 54–62)
Glucagonoma	Necrolytic Migratory Erythema (NME) (55–90%) Weight loss, hyperglycemia or overt diabetes Anemia, glossitis, stomatitis, cheilitis Diarrhea, altered bowel habits, general cachexia Venous thromboembolic events Hypoaminoacidemia (77)	Superficial Necrolytic Dermatitis (hepatocutaneous syndrome) Painful, erythematous, crusted, ulcerative lesions Affects: abdomen, inguinal region, distal limbs, paw pads, mucocutaneous junctions Lethargy, anorexia, vomiting Progressive weight loss Many develop diabetes mellitus and concurrent hepatopathy Chronic liver dysfunction Chronic, debilitating progression despite supportive care (24, 50–53)
Pancreatic NETs (Non-Functional)	Silent until mass effect size or metastasize (late diagnosis) Fatigue, weight loss, anorexia Abdominal pain, hepatomegaly, ascites (73, 83)	Nonspecific, progressive signs Vomiting, anorexia/hyporexia, abdominal distension Weight loss, lethargy, ascites Physical exam: dehydration, cachexia, pale mucous membranes, hypothermia (advanced) Lab: azotemia, anemia, hypoalbuminemia, elevated liver enzymes Cats: metastatic dissemination is common at diagnosis with hepatic/peritoneal involvement (20, 37, 39)
Paraneoplastic Hypoglycemia (IGF-II)	Rare, occurs with some non-islet cell tumors Hypoglycemia with inappropriate insulin levels (83, 84)	Dogs: Episodic weakness, collapse, tremors, seizures, altered mentation Triggered by fasting, exercise, stress or excitement Recurrent neuroglycopenic events with low/normal insulin concentrations (40, 41)
Gastric Carcinoids		Chronic, persistent vomiting, anorexia, weight loss, lethargy Advanced disease: weakness, ataxia, cough, anemia, hypoalbuminemia Gastric/metastatic lesions on ultrasonography (28, 29)
Hepatic/Hepatobiliary NETs		Anorexia, weight loss, vomiting, lethargy, hepatomegaly Extrahepatic: icterus and cholestasis or hepatocellular injury Paraneoplastic syndromes: hypoglycemia with seizure Rare, ectopic hormone secretion (hypercortisolism, persistent hypokalemia, episodic myopathy) Gallbladder: vomiting, hematemesis, melena, progressive weight loss, abdominal pain, lethargy, anemia, elevated liver enzymes (19, 30, 31, 63–67)
Intestinal Neuroendocrine Tumors		GI hemorrhage, chronic enteropathy, melena, hematemesis Chronic anemia, lethargy, anorexia, vomiting Intermittent diarrhea, progressive emaciation Advanced disease: metastatic spread, systemic clinical decline (32, 33)
Paragangliomas		Clinically silent or associated with excess catecholamine Systemic hypertension, weakness, mild hepatobiliary dysfunction (68, 69)
Cardiac Neuroendocrine Tumors		Secondary to mass effect on cardiac chambers Cough, tachypnea, exercise intolerance, weakness Progressive right-sided congestive heart failure, ascites, respiratory compromise (70)
Carcinoid/GEP-NETs (Functional)	Carcinoid Syndrome: Episodic flushing (head/neck, precipitated by stress, foods, alcohol, hot beverages) Diarrhea, abdominal cramping, wheezing Associated malabsorption Advanced/metastatic (liver): fibrosis of right-sided cardiac valves (tricuspid regurgitation, pulmonic stenosis) (80–82)	Reported in GI NETs but rarely documented Classic carcinoid syndrome manifestations (flushing, bronchospasm, valvular disease) are poorly or rarely described Suggests differences in metabolism of vasoactive mediators or physiological sensitivity (84)
General Non-Functional NETs	Fatigue, weight loss, anorexia, abdominal discomfort May remain asymptomatic for prolonged periods (83, 84)	Similar nonspecific systemic manifestations Delayed diagnosis due to silent growth (73)

Carcinoid tumors in human gastrointestinal NETs have partial parallels with gastrointestinal-derived NETs in companion animals. However, the classic hormonal syndrome (flushing, bronchospasm, valvular heart disease) appears to be poorly or rarely described in animals, indicating that physiological differences in the metabolism of vasoactive mediators, or differences in sensitivity, may prevent the complete manifestation of the syndrome as it occurs in humans. This suggests that, although animals with carcinoids/NETs may be useful for studying structural, invasive, or digestive aspects of the tumor, they have limitations in fully modeling human carcinoid syndrome, especially its hormonal and paraneoplastic components (84). In addition to physiological differences in the metabolism of vasoactive mediators, factors such as the shorter lifespan of companion animals or the frequency of early euthanasia following a cancer diagnosis may limit the full clinical manifestation of these chronic hormonal syndromes.

## 6 Imaging methods

### 6.1 Animals

#### 6.1.1 Insulinoma

A wide range of imaging techniques has been applied to canine (and feline) insulinomas, including transabdominal ultrasound, contrast-enhanced ultrasound (CEUS), contrast-enhanced CT (CECT) with multiple phases, CT angiography (CTA), single-photon emission CT (SPECT) with somatostatin-receptor tracers, PET–CT, and somatostatin-receptor scintigraphy; these modalities are variably sensitive for primary and metastatic disease and are commonly used for preoperative staging (85).

CECT frequently demonstrates arterial hyperenhancement of insulinomas, although this feature is not uniform. A quadruple-phase CECT series found avid arterial enhancement to be the predominant pattern in both primary tumors and presumed metastases, with the late arterial phase revealing the most conspicuous nodules. Hepatic and nodal lesions were commonly identified on arterial-phase images, although sampling showed a mixture of metastatic and benign findings. These data support the inclusion of early arterial imaging when performing multiphasic CT for suspected insulinoma (86).

Dual-phase CTA has also detected lesions not visible on conventional ultrasonography and correlated well with surgical and histopathological findings in small case series; arterial-phase imaging was critical to lesion conspicuity in those reports (87).

Comparative studies report heterogeneous performance across modalities: in one series, CT identified more primary tumors than ultrasound or SPECT but produced false-positive findings and missed some metastases; intraoperative exploration and palpation remain important adjuncts (88). In a larger retrospective cohort, CECT detected the majority of insulinomas but correctly localized the lesion within the pancreas in only about half of cases, with no clear superiority of single-, double- or triple-phase protocols for localization (85).

Magnetic resonance imaging (MRI) and MR cholangiopancreatography (MRCP) have been adapted from human practice to veterinary patients. Protocols typically include T2-weighted sequences (with and without fat suppression), T1-weighted pre- and post-contrast sequences, and high-resolution T2-weighted sequences for ductal visualization after secretin stimulation in some reports. MRI identified abnormal islet tissue and metastases in a small series, though signal characteristics varied (T2 hyperintensity and variable post-contrast T1 signal), reflecting differences in tumor composition (hemorrhage, stroma, necrosis). These early results indicate MRI/MRCP’s potential for non-invasive ductal and parenchymal assessment (89, 90).

Molecular and functional imaging yield mixed results: 18F-FDG PET–CT has shown inconsistent avidity for canine insulinomas, limiting its staging utility, likely because of variable tumor biology, size, and vascularity (91). Somatostatin-receptor imaging with 111In-pentetreotide (OctreoScan) may detect some insulinomas, but it is not uniformly localizing and can fail to lateralize intrapancreatic lesions (92).

In cats, ultrasound descriptions of insulinoma are limited but include well-defined hypoechoic pancreatic nodules; CEUS has detected additional small lesions not apparent on conventional ultrasound, leading to successful partial pancreatectomy (93, 94).

CEUS increases lesion conspicuity in many cases and can help differentiate tumor types: some series report variable transient hyperenhancement patterns for insulinomas, while exocrine adenocarcinomas tend to appear hypovascular and hypoechoic. Overall, CEUS can improve detection and provide perfusion information that complements CT and MRI (95, 96).

#### 6.1.2 Glucagonoma

Ultrasound may reveal a focal pancreatic mass in cases of glucagonoma, although reports are limited, and diagnosis depends on histopathology and endocrine testing (50).

#### 6.1.3 Gastrinoma

Gastrinomas are diagnostically and therapeutically challenging. Somatostatin analogue scintigraphy (111In-pentetreotide / octreotide) has identified multiple intra-abdominal foci consistent with pancreatic and hepatic disease in canine cases and, when combined with biochemical testing and histology, can confirm the diagnosis. Cross-sectional and ultrasound imaging may detect pancreatic or duodenal masses or hepatic metastases, but localization can be difficult and may require exploratory surgery (56, 58, 59, 97).

#### 6.1.4 Gallbladder NET

A 13-year-old neutered female Keeshond cross presented with a 1.5-year history of melena, anemia, hematemesis, vomiting, and persistent elevations in serum hepatic enzymes. Abdominal ultrasonography demonstrated a hyperechoic intraluminal gallbladder mass; color-flow Doppler identified a distinct linear vascular pattern within the lesion. These findings highlight the value of combining color-flow Doppler with conventional greyscale ultrasound when echogenic material is seen within the gallbladder, since assessment for intralesional blood flow may alter diagnostic and surgical planning (98).

#### 6.1.5 Intestinal NET

In an 8-year-old Labrador Retriever with progressive anorexia, constipation and depression, computed tomography (CT) showed invagination of the cecum into the ascending colon with a small, strongly arterial-enhancing cecal mass acting as the lead point. Dual-phase CT demonstrated a markedly hyperattenuating outer cecal layer (≈306 Hounsfield units) and a 7.5 × 8 mm intramural mass with intense arterial enhancement (≈310 HU) that became less conspicuous in the venous phase (≈120 HU). Adjacent mesenteric lymphadenopathy was also noted. These images underscore the utility of dual-phase CT to detect gastrointestinal tumors that serve as lead points for intussusception in older dogs (99).

#### 6.1.6 Paraganglioma

Paragangliomas, rare neuroendocrine tumors of paraganglionic tissue, may be malignant and therefore warrant precise characterization. In a reported case, CT revealed an irregular, mild contrast-enhancing mass abutting both adrenal glands with displacement of adjacent vessels, findings that aided localization and surgical planning (69).

#### 6.1.7 Hepatic neuroendocrine carcinoma

A 4-year-old spayed French bulldog with anorexia, lethargy, and vomiting had an abdominal ultrasound that showed multiple coalescing hyperechoic hepatic nodules. Single-phase contrast CT demonstrated heterogeneous hyperattenuating liver parenchyma with multiple hypoattenuating focal lesions on post-contrast images. Laparoscopic biopsy and immunohistochemistry confirmed primary hepatic neuroendocrine carcinoma. When ultrasound shows multiple hyperechoic nodules and post-contrast CT reveals hypoattenuating hepatic lesions, PHNEC should be included in the differential diagnosis (100).

### 6.2 Humans

Neuroendocrine tumors (NETs) are a heterogeneous and infrequent group of neoplasms that most commonly arise within the gastroenteropancreatic tract. At presentation, approximately half of patients have locoregional spread and roughly 27% harbor distant metastases; because stage directly informs therapeutic strategy, precise and timely imaging is fundamental to patient management (101).

Contemporary practice demands modality selection that reflects both tumor phenotype (for example, gastrinoma versus insulinoma) and histological grade (well versus poorly differentiated). Advances in cross-sectional and functional imaging, including dual-energy CT, diffusion-weighted MRI, liver-specific MR contrast agents, and novel nuclear tracers, have expanded the radiologist’s capability to characterize disease and to guide multidisciplinary decision making (102).

Conventional luminal studies retain a role in selected presentations. Barium studies and enteroclysis may reveal submucosal filling defects, polypoid or ulcerating masses, or secondary signs such as luminal narrowing and abnormal mucosal folding from infiltrative ileal disease. Enteroclysis is more sensitive than a standard small-bowel series for primary tumor detection, and, in some series, these fluoroscopic techniques outperform cross-sectional imaging for small intestinal primaries. Modern CT techniques, however, lessen this gap when optimized with intraluminal fluid, rapid intravenous contrast injection, and multiplanar reconstructions (103).

CT remains the rapid, robust first-line cross-sectional modality for patients with abdominal symptoms or suspected small, functional pancreatic NETs (PNETs). Contemporary scanners permit thin-section, multiphasic acquisitions and multiplanar reformats; emerging low-kilovoltage and multispectral techniques may further improve lesion detection (102).

The recommended protocol for suspected PNET is a dedicated, multiphasic pancreatic CT with an arterial phase (approximately 45–50 s after contrast bolus) and a portal venous phase (≈70 s). PNETs are classically well-circumscribed, solid lesions that demonstrate arterial hyperenhancement, and they may be multifocal throughout the gland. Such a tailored pancreatic protocol maximizes the likelihood of detecting small, hypervascular tumors (104).

In staging and surveillance, CT’s high temporal and spatial resolution, particularly when arterial-phase imaging is included, is complemented by MRI’s superior soft-tissue contrast for parenchymal organs. On CT and MRI, functioning PNETs typically appear as small, well-defined lesions with intense, homogeneous arterial enhancement; lesions with abundant fibrous stroma may instead show delayed enhancement, and some tumors present as cystic, complex cystic–solid, or calcified masses. Non-functioning PNETs are generally larger and demonstrate more heterogeneous and less intense enhancement (101).

Reported sensitivities for CT are variable but generally favorable when multiphasic techniques are employed. A consensus review estimated mean sensitivity at 73% and specificity at 96% for CT; for small functional tumors such as insulinomas, arterial-phase imaging markedly outperforms portal venous imaging (arterial-phase sensitivities reported in the 83–88% range, versus 11–76% in the portal phase), with some series showing near-complete detection on optimized late arterial (pancreatic parenchymal) imaging (102, 105, 106).

Primary hepatic NETs (PHNETs) most commonly present as multiple, well-circumscribed, heterogeneously enhancing lesions on CT and conspicuous T2-bright nodules on MRI; arterial-phase hyperenhancement with variable portal-phase washout and diffusion restriction are typical. Radiological appearance correlates with histological grade: grade-1 tumors tend to be solitary, rapidly enhancing nodules, whereas higher-grade lesions are more often multifocal with necrosis, hemorrhage, and reduced apparent diffusion coefficient values. Digital subtraction angiography classically demonstrates multiple hypervascular staining lesions in the arterial phase (107, 108).

Endoscopic ultrasound (EUS) has a pivotal role when small functioning pancreatic lesions or multifocal disease are suspected. EUS increases sensitivity relative to cross-sectional imaging for subcentimeter lesions and enables targeted tissue sampling, though its yield is operator dependent and best used as part of a complementary imaging pathway (109).

Functional imaging is indispensable for comprehensive staging and for theranostic patient selection. Somatostatin-receptor PET/CT (for example, ^68Ga-labelled analogues) has superior sensitivity to conventional SRS and SPECT and frequently alters management by revealing occult primaries or additional metastatic sites; it is now a mainstay in contemporary NET algorithms. ^18F-FDG PET/CT remains valuable for high-grade or poorly differentiated tumors, offering prognostic information and improved detection of aggressive disease that may be somatostatin-receptor-negative. The expanding radiopharmaceutical armamentarium, encompassing receptor-targeted and metabolism-targeted tracers, underpins a multimodal approach that integrates morphology, perfusion, and molecular phenotypes (101, 110, 111).

### 6.3 Comparison

Imaging strategies for neuroendocrine tumors in both domestic animals and humans share several core principles but also reflect important species-specific differences in clinical application and diagnostic performance (Table 4). In human medicine, multiphasic contrast-enhanced CT and MRI remain foundational for anatomical characterization, staging, and evaluation of small lesions or visceral metastases, and are often supplemented by functional imaging with somatostatin receptor PET using ^68Ga-labelled analogues. This hybrid imaging approach significantly enhances lesion detection beyond what morphology alone can achieve and directly influences clinical management and therapeutic selection. Studies in humans have demonstrated that functional imaging, such as SSR-PET/CT can provide additional diagnostic information in a substantial proportion of cases, particularly for small or occult metastases, and that novel hybrid modalities like PET/MRI offer the potential for comprehensive anatomical and molecular assessment with lower radiation exposure.

**Table 4:** Imaging modalities comparison (humans vs. animals).

Syndrome/Tumor type	Humans	Animals (dogs/cats)
First-Line Imaging	Multiphasic Contrast-Enhanced CT (pancreatic protocol) Arterial phase: ~45–50 s Portal venous phase: ~70 s (102, 104)	Transabdominal ultrasound (primary accessibility) Contrast-enhanced CT is increasingly used (85)
CT Protocol & Technique	Dedicated multiphasic pancreatic CT with thin sections Multiplanar reformats Low-kilovoltage and multispectral techniques emerging Arterial phase critical for small hypervascular tumors (102, 104)	Dual-phase CTA or quadruple-phase CECT Arterial phase essential for lesion detection The early arterial phase is most conspicuous for nodules (85, 86)
CT Sensitivity & Specificity	Mean sensitivity: 73%; Specificity: 96% Arterial vs. portal phase for small functional tumors: Arterial: 83–88% Portal: 11–76% (102, 105, 106)	Variable across studies CECT identifies the majority of insulinomas Correct localization within pancreas: ~50% of cases Heterogeneous performance across CT protocols (85–88)
CT Enhancement Pattern	Functioning PNETs: small, well-defined, intense homogeneous arterial enhancement Non-functioning PNETs: larger, heterogeneous, less intense enhancement Some show delayed enhancement (abundant fibrous stroma) or cystic/complex features (101)	Avid arterial enhancement predominant pattern Late arterial phase reveals the most conspicuous nodules Hepatic and nodal lesions are commonly identified on arterial-phase images (85, 86)
Ultrasound (US)	Retained role in selected presentations Less sensitive than CT for small primaries (100, 103)	Well-defined hypoechoic pancreatic nodules in cats CEUS increases conspicuity; may detect small lesions not apparent on conventional US Useful for differentiating tumor types (93–96)
Contrast-Enhanced Ultrasound (CEUS)	Limited routine uses in human practice (93–96)	Transient hyperenhancement patterns Helps differentiate insulinomas from adenocarcinomas Improves detection and provides perfusion information (85–88)
MRI/MRCP	Excellent soft-tissue contrast Complementary to CT for parenchymal organs Superior for detecting small lesions and metastases (89, 90)	Adapted protocols from human practice T2-weighted sequences (with/without fat suppression) T1-weighted pre/post-contrast Secretin-stimulated MRCP in selected cases Identifies abnormal islet tissue and metastases Variable signal characteristics (89, 90)
Functional Imaging: Somatostatin Receptor	Ga-labeled analogues now standard Superior sensitivity to conventional SRS and SPECT Reveals occult primaries and additional metastatic sites Alters management (101, 110, 111)	Somatostatin receptor scintigraphy (111In-pentetreotide/OctreoScan) May detect some insulinomas, but not uniformly localizing Limited availability Not standard outside research settings (92)
18F-FDG PET/CT	Valuable for high-grade/poorly differentiated tumors Offers prognostic information Detects aggressive disease, often somatostatin-receptor-negative (101, 110, 111)	Inconsistent avidity for canine insulinomas Limited staging utility Variable tumor biology, size, and vascularity (91, 92)
Endoscopic Ultrasound (EUS)	Pivotal role for suspected small functioning lesions or multifocal disease Increased sensitivity vs. cross-sectional imaging for subcentimeter lesions Enables targeted tissue sampling Operator-dependent (109)	Limited adoption in veterinary practice Not standard outside specialized centers (50, 93, 94)
Conventional Luminal Studies	Barium studies, enteroclysis: reveal submucosal filling defects, polypoid/ulcerating masses Secondary signs: narrowing, abnormal folds Enteroclysis is more sensitive than the small bowel series for primaries Some series outperform cross-sectional imaging for small intestinal primaries (103)	Not commonly used in veterinary practice (98, 103)
Hepatic NETs Imaging	Multiple well-circumscribed, heterogeneously enhancing lesions on CT T2-bright nodules on MRI Arterial hyperenhancement with variable portal-phase washout Diffusion restriction is typical Grade correlates with appearance (107, 108)	Multiple hyperechoic hepatic nodules on US Single-phase CT: heterogeneous hyperattenuating liver with multiple hypoattenuating focal lesions post-contrast Laparoscopic biopsy for confirmation (100)
Staging and Surveillance	CT and MRI: high temporal/spatial resolution Multiphasic technique maximizes detection Integration with functional imaging critical for comprehensive staging (101, 102, 105–109)	CT and ultrasound: primary modalities for mass identification and staging Intraoperative exploration/palpation remains an important adjunct (85, 88, 93–96)

In veterinary practice, the relative rarity of neuroendocrine tumors and variability in presentation have historically limited the routine use of advanced functional imaging. Domestic species frequently rely on ultrasound and CT as primary modalities due to their accessibility, speed, and capability to identify mass lesions, particularly for pancreatic tumors such as insulinomas. Emerging reports underscore the utility of contrast-enhanced ultrasonography and multiphasic CT for detecting hypervascular lesions and staging disease. While nuclear medicine techniques such as somatostatin receptor scintigraphy or PET are well established in human NET imaging, their adoption in veterinary medicine remains restricted by limited availability and higher cost, and they are not yet standard practice outside of research settings.

## 7 Treatment

### 7.1 Animals

#### 7.1.1 Intestinal neuroendocrine tumors

A canine intestinal carcinoid was managed with complete surgical excision followed by adjuvant carboplatin chemotherapy (300 mg/m^2^ every 3 weeks, 4 cycles). Treatment was well tolerated, without reported toxicity, and resulted in durable remission exceeding 18 months. This represents the first documented case of long-term disease control using adjuvant chemotherapy in canine intestinal neuroendocrine tumors (112).

#### 7.1.2 Glucagonoma

A dog with metastatic pancreatic glucagonoma and necrolytic migratory erythema was treated medically with subcutaneous octreotide. Therapy induced rapid and marked improvement of cutaneous and systemic signs, with dose adjustments required to balance efficacy and appetite. Interruption of treatment led to prompt relapses, while reinitiation restored clinical control. Although survival was limited by progressive metastatic disease, octreotide provided effective symptomatic management (25).

#### 7.1.3 Hepatic neuroendocrine carcinoma

A primary hepatic neuroendocrine carcinoma in a dog was treated with high-dose doxorubicin followed by metronomic cyclophosphamide and meloxicam. The regimen was well tolerated and achieved prolonged disease stabilization, with survival of approximately 15.5 months. This case represents the first report describing this chemotherapeutic approach for primary hepatic neuroendocrine carcinoma in dogs (113).

### 7.2 Pancreatic NETs

#### 7.2.1 Pancreatic neuroendocrine tumors

Recurrent pancreatic carcinoid-associated ductal obstruction in a cat was successfully treated with en bloc resection and complex reconstructive surgery, followed by postoperative toceranib. The procedure was well tolerated, without major complications, and resulted in a favorable medium-term outcome, supporting aggressive surgical management when anatomically feasible (114).

#### 7.2.2 Gastrinoma

In feline gastrinoma, combined surgical excision and medical therapy with omeprazole and toceranib resulted in long-term survival exceeding 35 months, although toceranib was discontinued because of hyporexia. In non-surgical cases, medical management with proton pump inhibitors and octreotide, guided by intragastric pH monitoring, achieved ulcer resolution and sustained palliation. These reports support acid suppression, with or without somatostatin analogues, as effective strategies when surgery is contraindicated (58, 59).

Medical management with omeprazole alone led to complete resolution of clinical signs and sustained remission over 2 years in a dog with Zollinger-Ellison syndrome, supporting proton pump inhibitors as first-line therapy for gastrinoma-associated acid hypersecretion (115).

Octreotide administration produced transient suppression of circulating gastrin concentrations. Combination therapy with acid suppressants and escalating octreotide doses enabled survival up to 14 months, indicating a supportive role for somatostatin analogues in refractory cases (97).

#### 7.2.3 Insulinoma

Surgical resection remains the treatment of choice for canine insulinoma and is associated with significantly longer survival than medical management alone, despite risks of pancreatitis and diabetes mellitus. Prognosis is influenced by disease stage, metastatic status, postoperative glycemic control, and tumor invasiveness (18, 116, 117).

Diazoxide effectively controlled hypoglycemia in approximately 70% of dogs, either as monotherapy or adjunctive treatment, although gastrointestinal, hematological, and renal adverse effects were reported. In cats unsuitable for surgery, diazoxide provided short-term glycemic control until death from unrelated disease (118, 119).

Dexmedetomidine suppressed insulin secretion and maintained euglycemia perioperatively with minimal cardiovascular effects in a case report. Glucagon infusions, including constant-rate infusion protocols, were consistently effective for rapid correction of refractory hypoglycemia and seizure control, even in cases resistant to conventional therapies (120–123).

Octreotide selectively suppressed insulin secretion in dogs with insulinoma, leading to increased plasma glucose without inhibiting counter-regulatory hormones, supporting its potential role in long-term medical management. Medetomidine similarly reduced insulin concentrations and increased glucose levels during anesthesia (124, 125).

Toceranib phosphate improved progression-free and overall survival in dogs with metastatic or recurrent insulinoma and was generally well tolerated. Streptozotocin demonstrated antitumor activity but was limited by variable efficacy and significant metabolic and renal toxicity (126–130).

Percutaneous ultrasound-guided radiofrequency ablation achieved consistent glycemic control and tumor size reduction with minimal complications, representing a viable alternative to surgery. Minimally invasive laparoscopic pancreatectomy and intraoperative glucose monitoring further improved surgical precision and long-term outcomes in selected cases (116, 131–133).

### 7.3 Humans

Treatment of NETs in humans is highly individualized and requires a multidisciplinary approach, resonating with the tumor’s heterogeneous biological activity (Table 5). In general, therapeutic strategies are guided by both patient and tumor background, including comorbidities, primary site, differentiation, grade, functional status, expression of somatostatin receptors (SSTR), and disease stage. The most widely used treatment options include somatostatin analogs (SSAs), surgical resection, liver-directed therapies, chemotherapy, immunotherapy, and peptide-receptor radionuclide therapy (PRRT) (134, 135).

**Table 5:** Treatment modalities comparison (humans vs. animals).

Syndrome/Tumor type	Humans	Animals (Dogs/Cats)
Surgical Resection	Localized/Well-Differentiated NETs: Preferred treatment Curative intent with complete resection and lymphadenectomy Even metastatic disease: cytoreduction beneficial (symptom control) (134–136)	Insulinoma: Primary treatment of choice Significantly longer survival than medical management alone Risks: pancreatitis, diabetes mellitus development Prognosis influenced by stage, metastatic status, glycemic control, invasiveness (18, 47, 116, 117)
Minimally Invasive Surgery	Laparoscopy: limited role, not standard (116, 131–133)	Laparoscopic pancreatectomy with intraoperative glucose monitoring Improved precision and outcomes in selected cases (116, 131–133)
Ablative Techniques	Embolization, thermal ablation Stereotactic body radiation therapy for hepatic metastases (134, 136)	Percutaneous ultrasound-guided radiofrequency ablation Consistent glycemic control and tumor size reduction Minimal complications Viable alternative to surgery (116, 131–133)
Somatostatin Analogs (SSAs)	First Line for SSTR+ tumors: Octreotide, Lanreotide (LA) 65% reduction in diarrhea (both agents) 72% reduction in flushing (Octreotide); 69% (Lanreotide) Carcinoid: CLARINET trial - extended-release Lanreotide enhances PFS Delays disease progression in SSTR+ tumors (Ki-67 < 55%) (134, 137–139)	Glucagonoma: Subcutaneous octreotide induces rapid improvement Gastrinoma: Omeprazole + octreotide guided by intragastric pH; achieves ulcer resolution Insulinoma: Octreotide suppresses insulin secretion, increases plasma glucose without inhibiting counter-regulatory hormones Potential for long-term medical management (25, 97, 115)
Insulin Suppression Agents	No standard in humans (134–136)	Diazoxide: ~70% effectiveness (monotherapy or adjunctive); GI, hematologic, renal adverse effects Dexmedetomidine: Perioperative insulin suppression with minimal cardiovascular effects Medetomidine: Reduces insulin, increases glucose during anesthesia Glucagon: Constant-rate infusion effective for refractory hypoglycemia and seizure control (118–125)
Proton Pump Inhibitors	Gastrinoma/Zollinger-Ellison: Effective control of acid hypersecretion Can be sole therapy (58, 59, 115)	Gastrinoma: Omeprazole alone - complete resolution of signs and sustained remission (2 + years) Combined with toceranib and octreotide in a feline case: >35 months survival (58, 59, 115)
Peptide Receptor Radionuclide Therapy (PRRT)	SSTR+ SSA-Refractory: 68Ga-DOTATATE, 177Lu-DOTATATE, 90Y-DOTATATE NETTER-1: ~22.7-month gain in time to deterioration Long-term bone marrow toxicity concern (135, 140, 141)	No reports found of usage in veterinary practice
Chemotherapy	Metastatic/Progressive G3 GEP-NETs: Capecitabine-Temozolomide (CAPTEM), 5-FU, Oxaliplatin CAPTEM shows better PFS vs. Temozolomide monotherapy (135, 145)	Intestinal Carcinoid: Adjuvant carboplatin (300 mg/m^2^ q3wks, 4 cycles); durable remission >18 months Hepatic NET Carcinoma: High-dose doxorubicin + metronomic cyclophosphamide + meloxicam; ~15.5 months survival (112, 113)
Targeted/Kinase Inhibitors	mTOR Pathway: Everolimus for progressive G1-G2 GI/pan-NETs; favorable PFS Tyrosine Kinase Inhibitors: Sunitinib for progressive disease; favorable PFS (135, 142–144)	Insulinoma: Toceranib phosphate improved PFS and overall survival in metastatic/recurrent cases Gastrinoma (Feline): Toceranib with omeprazole/octreotide; >35 months survival (limited by hyporexia) Streptozotocin: Antitumor activity but limited by variable efficacy and significant toxicity (58, 59, 126–130)
Immunotherapy	Emerging role in select cases; limited data compared to traditional approaches (134, 135)	Apparently not yet established in veterinary oncology for NETs
Combination/Multimodal	NCCN/ASCO: Multidisciplinary strategy based on grade, stage, functional status, SSTR expression, Ki-67 index Hepatic Metastases: Liver-directed therapies (debulking, embolization, ablation, SBRT) improve long-term survival (134–136)	Pancreatic Carcinoid (Cat): En bloc resection + reconstructive surgery + postoperative toceranib Surgery + chemotherapy; Surgery + medical management; Medical management (acid suppression + SSA) when non-surgical (116, 126, 131–133)

Well-differentiated, localized NETs are preferentially managed with complete surgical resection of the primary tumor, with or without lymphadenectomy. Selected patients with metastatic disease may also benefit from tumor cytoreduction, not only by reducing tumor burden, but also by providing symptomatic control, particularly in those with functional tumors. The 2021 NCCN guidelines on neuroendocrine and adrenal tumors management consider slow-growing, SSTR-positive tumors – typically with a Ki-67 index below 55% – to be favorable for resection, even in the setting of advanced disease. Moreover, liver-directed therapies are emphasized, ranging from surgical debulking of hepatic metastases to embolization, thermal ablation, and stereotactic body radiation therapy. Liver-directed interventions have been associated with improved long-term survival and are recommended whenever feasible (134, 136).

In functional and SSTR-positive tumors, SSAs play a pivotal role in symptom control and in delaying disease progression. The 2026 ASCO guideline on symptom management of well-differentiated GI-NETs places long-acting SSAs as first-line therapy in patients with carcinoid syndrome_._ A systematic review by Hofland et al. demonstrated a 65% reduction in diarrhea among patients treated with either Octreotide or Lanreotide, as well as a 72% reduction in flushing with Octreotide and 69% with Lanreotide. Additionally, the phase III CLARINET trial revealed that extended-release Lanreotide notably enhances progression-free survival (PFS) (134, 137–139)_._

In the context of targeted therapies, PRRT has emerged as an option for SSTR-positive, SSA-refractory cases, with significant results in symptom management, as well as an estimated gain of 22.7 months in time of deterioration, as shown in the phase III NETTER-1 trial_._ As per the 2023 ASCO guideline on systemic therapy for metastatic GEP-NETs, SSAs are still recommended concomitantly with PRRT in patients with functional tumors. Despite its benefits, the guideline highlights a potential increased risk of long-term bone marrow toxicity after PRRT treatment (135, 140, 141).

From an immunotherapy standpoint, mTOR pathway inhibitors (e.g., Everolimus) and tyrosine kinase inhibitors (e.g., Sunitinib) are representative of progressive disease, yielding favorable PFS outcomes in patients with GI and pan-NETs_._ Everolimus is an option for patients with nonfunctional, G1-G2 metastatic SSTR-negative tumors or SSTR-positive tumors who are not eligible for PPRT due to concerns regarding hematologic toxicity. Recommendations from the 2023 ASCO guideline included PRRT, Everolimus, Sunitinib, or chemotherapy as a second or later-line option for patients with low-grade pan-NETs (135, 142–144).

Chemotherapy regimens (e.g., capecitabine and temozolomide [CAPTEM], fluorouracil, leucovorin, oxaliplatin) are generally indicated for patients with higher disease volumes and/or significant tumor-related symptoms, particularly in G3 GEP-NETs and pan-NETs. In a study comparing CAPTEM versus Temozolomide monotherapy in pan-NETs, patients receiving CAPTEM exhibited better PFS percentages. However, objective response rates were statistically similar between the two groups, with fewer reported adverse events in the Temozolomide monotherapy arm (135, 145).

## 8 Conclusion

Neuroendocrine tumors in companion animals and humans share overlapping presentations. Pancreatic neuroendocrine tumors, particularly insulinomas, represent the clearest comparative interface, with similar hormone-mediated syndromes, conserved neuroendocrine morphology and expression of synaptophysin and chromogranin A support the use of shared immunohistochemical panels across species. In human medicine, multiphasic CT, MRI, and somatostatin receptor-based imaging are central to evaluation, whereas veterinary practice relies mainly on ultrasound and contrast-enhanced CT. Surgery remains the cornerstone for localized disease in both contexts, complemented by medical strategies aimed at controlling hormone excess and tumor progression. While veterinary evidence is largely derived from retrospective series and case reports, human management is increasingly standardized through international guidelines and structured therapeutic pathways.

Although this review supports companion animals as biologically relevant comparative models, its conclusions are limited by a single-database search strategy and the inherent constraints of a literature-review design.
